# Revisiting the molecular landscape of Rosai-Dorfman disease: insights from whole exome sequencing of Saudi patients

**DOI:** 10.3389/fonc.2025.1556830

**Published:** 2025-05-08

**Authors:** Sarah Binhassan, Manar Samman, Yazeed Al-Sheikh, Wafaa Alshakweer, Tahani Alhalouli, Najd Alshamlan, Manal Abudawood

**Affiliations:** ^1^ Department of Clinical Laboratory Sciences, College of Applied Medical Sciences, King Saud University, Riyadh, Saudi Arabia; ^2^ Central Research Laboratory, King Saud University, Riyadh, Saudi Arabia; ^3^ Molecular Pathology (Genetics) Section, Pathology and Clinical Laboratory Medicine Administration, King Fahad Medical City, Riyadh, Saudi Arabia; ^4^ Department of Pathology and Laboratory Medicine, King Fahad Medical City, Riyadh, Saudi Arabia; ^5^ Department of Pathology and Laboratory Medicine, King Abdullah bin Abdulaziz University Hospital, Riyadh, Saudi Arabia

**Keywords:** Rosai-Dorfman disease, whole exome sequencing, molecular markers, *BRCA1* mutation, genetic landscape

## Abstract

Rosai-Dorfman disease (RDD) has traditionally been viewed as a reactive histiocytic disorder, defined by its unique clinical features and immunophenotype. However, recent genetic studies suggest a more complex molecular landscape, challenging the notion of RDD as solely reactive and hinting at a possible neoplastic component. Mutations in MAPK/ERK pathway genes, such as *KRAS* and *MAP2K1*, have been observed in up to 33% of cases. Additional genetic alterations in cell cycle regulation, DNA repair, and other processes, along with low-frequency *BRAF* mutations, further emphasize this complexity. To better understand the molecular basis of RDD and enhance diagnostic precision, we conducted whole exome sequencing (WES) on seven Saudi patients with RDD, comparing their genetic profiles with existing literature. While no kinase driver mutations were detected, our analysis revealed thirteen distinct mutations. Recurrent mutations were observed in *CD207* and *TDG*, each found in six patients. *CD207* is linked to antigen processing, while *TDG* is associated with DNA repair. *MUC4* and *PDS5A* mutations, related to cell cycle regulation, were each identified in three patients. *DNMT3A* mutation, affecting DNA methylation, was found in two patients. Single mutations were observed in *BRCA1*, *LATS2*, *ATM*, *USP35*, and *CIC*, associated with DNA repair, the ubiquitin proteasome pathway, and transcriptional regulation respectively. These findings offer insights into the genetic makeup of RDD, revealing candidate genes and expanding our understanding of the disease’s molecular complexities. By uncovering these genetic markers, this study contributes to the ongoing efforts to develop more accurate diagnostic tools and refine the classification of RDD, paving the way for improved patient care and disease management.

## Introduction

Rosai–Dorfman disease (RDD) is a rare histiocytic proliferative disorder that was characterized by Destombes in 1965 and later by Rosai and Dorfman in 1969. They described it as sinus histiocytosis with massive lymphadenopathy (1, 2). RDD clinically manifests as painless bilateral massive cervical lymphadenopathy associated with fatigue, weight loss fever, and night sweats (2, 3). The diagnoses require the presence of large histiocytic cells displaying characteristic features of abnormal S100^+^, fascin^+^, CD68^++^, CD14^+^, HLA-DR^+^, CD163^+^, CD1a^-^, and CD207^-^macrophages. The involved tissue usually contains numerous polyclonal plasma cells. The sinuses of lymph nodes mainly contain large S100^+^ histiocytes, while the cortex is infiltrated by abundant plasma cells and activated B cells (4, 5).

The etiology of RDD is not very well understood. Studies of viral agents, such as human herpesvirus 6, parvovirus B19 and Epstein-Barr failed to prove them as predisposing factors to the disease (6). Different gene mutations associated with RDD have been described recently. Mutations involving the MAPK/ERK pathway, i.e., *KRAS* and *MAP2K1* were found in up to 33% of cases with RDD (7). A recent study on 17 RDD patients revealed multiple gene alterations. It showed variable percentages of kinase driver mutations including *KRAS, MAP2K1, NRAS*, *ARAF* and *CSF1R* (8). Additionally, genes involved in different cell functions were mutated. These included cell cycle regulation genes (*PDS5A*, *MUC4)*, transcriptional regulation genes (*CIC*, *INTS2*, *SFR1*, *BRD4*, *PHOX2B*), DNA mismatch repair genes (*ERCC2*, *LATS2*, *BRCA1*, *ATM*), intracellular trafficking (*SNX24*), and the ubiquitin proteasome pathway gene (*USP35*) (8). A low frequency of *BRAF* mutations in cases of RDD were demonstrated in different studies. For example, deletion in exon 12 of *BRAF*, V600E, Y472C and R188G were reported (9–11). Some mutations in *SEC62, PIK3R2, PIK3CA, TLR8, FCGBP, VCL*, *EGFR*, and *ERBB2* were also found (11). Moreover, alterations in genes that are commonly mutated in lymphoid and myeloid malignancies i.e. *DNMT3A*, *TET2*, *MLL4*, *NF1*, *ASXL1*, and *ALK* were observed in RDD patients (11, 12).

Many of these molecular findings indicate that part of RDD is a malignant process rather than a reactive disorder. Although some of the demonstrated gene mutations are associated with malignancies, their tumorigenic role in RDD has yet to be studied. Also common occurrence of these or other mutations in RDD patients need to be further explored. In this study, seven patients from King Fahd Medical City (KFMC) diagnosed with RDD were selected for whole exome sequencing (WES). The study compared mutations identified in RDD patients through WES with those reported in the existing literature. This combined analysis aimed to improve the molecular understanding of the disease and aid in developing more accurate diagnostic tools in the future.

## Materials and methods

### Clinical data

The clinical data and specimens were collected under appropriate Institutional review board (IRB) approvals from KFMC institutional ethical committee, Riyadh.

### DNA isolation

Genomic DNA of RDD specimens was isolated from Formalin-Fixed Paraffin-Embedded (FFPE) tissue sections using the QIAamp DNA FFPE Tissue Kit (QIAGEN, Hilden, Germany) according to the manufacturer’s protocol. Briefly, deparaffinization was performed with xylene and ethanol washes to remove paraffin. Proteinase K digestion in Buffer ATL lysed the cells and released DNA. The lysate was then purified using QIAamp MinElute spin columns to remove contaminants. Finally, purified DNA was eluted in an appropriate volume of Buffer ATE for sequencing. The DNA concentration was assessed by spectrophotometry using the Nanodrop ND 1000 (NanoDrop, UNISCIENCE, USA).

### Whole Exome Sequencing (WES)

RDD specimens of pathologically confirmed cases were included. WES was performed on DNA extracted from FFPE tissue. The samples were sent to an external institution for Whole Exome Sequencing (WES) using next-generation sequencing. Testing was performed according to the institution’s laboratory standard protocol. Briefly, target regions were enriched using DNA capture probes, after which genomic DNA was enzymatically fragmented. These target regions include mitochondrial genome in addition to approximately 41 Mb of the human coding exome (targeting > 98% of the coding RefSeq from the human genome build GRCh37/hg19). To allow at least 20x coverage depth for > 98% of the targeted bases, the generated library was sequenced on an Illumina platform. The investigation of relevant variants was performed using Qiagen Clinical Insight (QCI) Variants (QIAGEN, Hilden, Germany), following the Standards and Guidelines for the Interpretation of Sequence Variants of the American College of Medical Genetics (ACMG) (13). According to these guidelines the sequence variants are categorized into five classes; i) pathogenic, ii) Likely pathogenic, iii) variant of unknown significance, iv) likely benign, v) benign.

## Results

There were seven patients with RDD, including 4 males and 3 females. The ages ranged from 38–66 years, with an average age of 48.4 years and a median age of 48 years (**Table 1**). The presenting symptom was a mass, with four patients presenting with brain masses and two with breast masses. One patient presented with a right facial mass.

**Table 1:** List of demographics, site of biopsy, associated findings, and outcomes in Saudi patients with Rosai-Dorfman disease (RDD).

**Patient No.**	**Age/gender**	**Site of biopsy**	**Associated findings**	**Outcome at Last Follow-up**
1	38/Male	Right facial mass	Reoccurrence of Cutaneous RDD	Calcified cystic liver lesion
				No recurrence of RDD
2	48/Male	Brain mass	None	T2DM
				Eplipsy
				No recurrence of RDD
3	66/Female	Right breast mass	Right breast carcinoma	T2DM
				No distant metastasis
				No recurrence of RDD
4	44/Female	Brain mass	None	Clinically stable
				No recurrence of RDD
5	42/Male	Brain mass	Parasagittal meningioma post debulking	No recurrence of RDD
6	50/Female	Right breast mass	None	T2DM
				No recurrence of RDD
7	51/Male	Brain mass	Meningioma	Daytime hypersomnolence
				No recurrence of RDD

T2DM, type 2 diabetes mellitus.

Among the patients with brain masses, one had a parasagittal meningioma post-debulking (Patient No.5) and another had an associated meningioma (Patient No.7). Patient No.1 with facial mass had reoccurrence of cutaneous RDD. Patient No.3, who presented with a right breast mass had right breast carcinoma (**Table 1**).

At the last follow-up, other conditions were observed in several patients. Three patients had type 2 diabetes mellitus (T2DM) (Patients No.2, 3, and 6). Patient No.2 also had epilepsy. Patient No.1 had a calcified cystic liver lesion. No distant metastasis was observed in Patient No.3 with breast carcinoma. Patient No.7 had daytime hypersomnolence. All patients showed no recurrence of RDD at the last follow-up (**Table 1**).

All seven cases were sent for sequencing to assess for genetic mutations. The DNA obtained from the FFPE tissue of Patient No.7 (**Table 1**) was highly degraded and the DNA sample quality was insufficient to perform the sequencing analysis. Therefore, there were sequencing results for six patients. The sequencing data were analyzed following the ACMG guidelines. The primary focus was on the pathogenic, likely pathogenic alterations and Variants of Uncertain Significance (VUS). Looking for the mutations that have already been reported in the literature. Kinase driver mutations i.e. *KRAS, MAP2K1, NRAS*, *ARAF* and *CSF1R* were not detected in all cases. However, WES revealed a spectrum of molecular alterations across various genes involved in key cellular processes (**Table 2**). A total of thirteen distinct mutations in this cohort were detected. The most frequently observed variants were found in the *CD207* and *TDG* genes, each exhibiting mutations in six patients. Specifically, *CD207* showed a (c.71 + 2dupG) duplication, associated with antigen processing and presentation to T cells, while *TDG* presented (c.292dupA) duplication, linked to DNA demethylation and DNA repair (**Table 2**).

**Table 2:** Frequency and spectrum of gene variants in **Saudi patients with** Rosai-Dorfman disease (RDD).

Number of mutation carriers	Gene function	Gene name	Gene variant
6	Antigen processing and presentation to T cells	*CD207*	c.71+2dupG
6	DNA demethylation/DNA repair	*TDG*	c.292dupA
3	Cell cycle regulation	*MUC4*	c.6075_6077dupATC
2	Cell cycle regulation	*PDS5A*	c.528-5dupT
2	DNA methylation	*DNMT3A*	c.126G>A
1	Cell cycle regulation	*PDS5A*	c.1088-13del
1	DNA mismatch repair	*BRCA1*	c.2157dupA
1	DNA mismatch repair	*LATS2*	c.1907_1908ins
1	DNA mismatch repair	*ATM*	c.1236-3dupT
1	DNA mismatch repair	*ATM*	c.3403-13dupA
1	Ubiquitin proteasome pathway	*USP35*	c.2351C>T
1	Transcriptional regulation	*CIC*	c.206G>A
1	Transcriptional regulation	*CIC*	c.302G>A

Three patients carried mutations in the *MUC4* gene, which is involved in cell cycle regulation, with the specific mutation being (c.6075_6077dupATC). Three patients demonstrated mutations in *PDS5A* associated with cell cycle regulation, two exhibited (c.528-5dupT) duplication and one had (c.1088-13del). Two patients showed *DNMT3A* linked to DNA methylation with (c.126G>A) mutation (**Table 2**).

Single mutations were observed in *BRCA1* (c.2157dupA), *LATS2* (c.1907_1908ins), *ATM* (c.1236-3dupT and c.3403-13dupA) all involved in DNA mismatch repair. Additional single mutations *USP35* (c.2351C>T), and *CIC* (c.206G>A and c.302G>A) associated with ubiquitin proteasome pathway, and transcriptional regulation, respectively. These findings highlight the diverse genetic landscape within this cohort, with mutations spanning critical pathways such as DNA repair, cell cycle control, transcription regulation and immune response.

Further analysis of individual patients identified a distinct pattern of mutations across various genes. To provide a more comprehensive understanding of the genetic landscape of RDD in the Saudi population, the Mean Allele Frequency (MAF), Homozygous allele frequency (HomAF), and Heterozygous allele frequency (HetAF) were derived from the KFMC database (**Table 3**). These allele frequencies represent a sample size of 4,564 individuals and 2.225 million variants from the Saudi population.

**Table 3:** Gene variants identified in six Saudi patients with RDD.

Patient No.	Gene name	Gene variant	Classification	Genotype	MAF	HomAF	HetAF
1	*CD207*	c.71+2dupG	VUS	hom	100%	100%	0%
	*TDG*	c.292dupA	P	het	43%	0%	43%
	*MUC4*	c.6075_6077dupATC	VUS	het	17.4%	0.4%	17%
	*DNMT3A*	c.126dupG	LP	het	N/A	N/A	N/A
	*PDS5A*	c.1088-13del	VUS	het	35%	10%	25%
2	*CD207*	c.71+2dupG	VUS	hom	100%	100%	0%
	*TDG*	c.292dupA	P	het	43%	0%	43%
	*LATS2*	c.1907_1908ins	LP	het	N/A	N/A	N/A
3	*CD207*	c.71+2dupG	VUS	hom	100%	100%	0%
	*TDG*	c.292dupA	P	het	43%	0%	43%
	*DNMT3A*	c.126dupG	LP	het	N/A	N/A	N/A
	*USP35*	c.2351C>T	VUS	het	0.09%	0%	0.09%
	*CIC*	c.206G>A	VUS	het	0.81%	0%	0.81%
4	*CD207*	c.71+2dupG	VUS	hom	100%	100%	0%
	*TDG*	c.292dupA	P	het	43%	0%	43%
	*MUC4*	c.6075_6077dupATC	VUS	het	17.4%	0.4%	17%
	*PDS5A*	c.528-5dupT	VUS	het	1.88%	0%	1.88%
5	*CD207*	c.71+2dupG	VUS	hom	100%	100%	0%
	*TDG*	c.292dupA	P	het	43%	0%	43%
	*BRCA1*	c.2157dupA	P	het	N/A	N/A	N/A
	*PDS5A*	c.1088-13del	VUS	het	35%	10%	25%
	*ATM*	c.1236-3dupT	LB	het	8%	1%	7%
	*ATM*	c.3403-13dupA	LB	het	27%	16%	11%
6	*CD207*	c.71+2dupG	VUS	hom	100%	100%	0%
	*TDG*	c.292dupA	P	het	43%	0%	43%
	*MUC4*	c.6075_6077dupATC	VUS	het	17.4%	0.4%	17%
	*CIC*	c.302G>A	VUS	het	N/A	N/A	N/A

MAF, mean allele frequency; HomAF, homozygous allele frequency; HetAF, heterozygous allele frequency; VUS, variant of unknown significance; P, pathogenic; LP, likely pathogenic, hom, homozygous; het, heterozygous. N/A indicates not available.

All six patients exhibited homozygous mutations in the *CD207* gene (c.71 + 2dupG), indicating a 100% HomAF within this cohort. This variant has a 100% MAF and 100% HomAF in the KFMC database. Similarly, all patients carried heterozygous pathogenic mutations in the *TDG* gene (c.292dupA) with a 100% HetAF in this cohort. The KFMC database shows a 43% MAF and 43% HetAF for this variant (**Table 3**).

The data also displayed VUS in *PDS5A* (c.1088-13del in Patients No.1 and No.5, c.528-5dupT in Patient No.4), *USP35* (c.2351C>T in Patient No.3), and *CIC* (c.206G>A in Patient No.3, c.302G>A in Patient No.6). The MAFs for these variants in the KFMC database ranged from 0.09% to 35% with a 100% HetAF in this cohort except for *CIC* (c.302G>A) unavailable data.

The *MUC4* gene (c.6075_6077dupATC) was found in three patients (Patients 1, 4, and 6) in a heterozygous state, corresponding to a 100% HetAF in this cohort. The MAF for this variant in the KFMC database is 17.4% and the HetAF is 17%.

Heterozygous likely pathogenic mutations in *DNMT3A* (c.126dupG) were identified in two patients (Patients No.1 and No.3). Patient No.2 had a heterozygous likely pathogenic mutation in *LATS2* (c.1907_1908ins), and Patient No.5 had a heterozygous pathogenic mutation in *BRCA1* (c.2157dupA). Allele frequencies for these mutations were not available in the KFMC database. In addition to the *BRCA1* mutation, Patient No.5 also harbored two likely benign variants in the ATM gene in a heterozygous state (c.1236-3dupT and c.3403-13dupA) with a 7% and 11% HetAF, respectively, at KFMC (**Table 3**). These findings highlight a consistent pattern of *CD207* and *TDG* mutations across all RDD patients in this cohort, alongside a variety of other gene alterations with varying classifications and frequencies within both the cohort and the broader Saudi population as represented by the KFMC database.

## Discussion

Taken the rarity of the Rosai-Dorfman disease, few studies explored its pathogenesis. The molecular studies of individual cases including first-generation or second-generation sequencing did not conclude on a common genetic lesion as a cause of RDD. In a study of 21 RDD cases, alterations in kinase driver genes such as *KRAS* and *MAP2K1* were found in four and three cases, respectively. Immunohistochemistry showed that patients with *MAP2K1* had p-ERK overexpression. The presence of mutations correlated with two factors: occurring in children (pediatric patients) and being located in the head and neck region. No alterations were identified in *ARAF, BRAF, PIK3CA*, or any other genes that were assessed in that study. They suggested that involvement of the MAPK/ERK pathway could explain the development of a subgroup of RDD. Therefore, this subgroup might be clonal due to kinase activation (7).

In another study six patient samples were sequenced to assess for mutations in cancer-related genes. Mutations in three MAPK-related genes i.e. *MAP2K, PTPN11*, and *NFT1* were identified in one case. This case also had mutations in *ASXL1* and *TET2*. Another case carried a mutation in *TIMP3*. Mutations in *KRAS* were found in two cases, where one of them also harbored *DNMT3A* mutation (12).

Other studies revealed *BRAF* (V600E) mutation in a child with both RDD and Langerhans’ cell histiocytosis (LCH), and pathogenic mutation in exon 12 of the *BRAF* gene in a patient with central nervous system involvement (10, 14). In addition to V600E and deletion in exon 12, *BRAF* Y472C and *BRAF* R188G mutations were demonstrated by other groups (9–11). A recent study supports the view that some RDD is monoclonal, where *NRAS* mutations were detected in 3/19 cases and *KRAS* mutation in 1/19 cases. However, none of the 19 cases showed *BRAF* or *MAP2K1* gene mutations (15).

The present study, involving genetic sequencing of six RDD cases, reveals genetic landscape distinct from the previously reported MAPK pathway alterations. A consistent finding was the presence of homozygous *CD207* (c.71 + 2dupG) and heterozygous pathogenic *TDG* (c.292dupA) mutations in all patients. Further analysis, utilizing the KFMC database, revealed these alterations as common polymorphisms within the Saudi population (**Table 3**). Due to this high population frequency, their role in specific RDD pathogenesis is therefore excluded. The prevalence of these variants explains their common existence throughout this six patient cohort. This finding highlights the importance of population-specific genetic databases in distinguishing disease-causing mutations from benign variants, thereby preventing misclassification of common variants as pathogenic drivers.

Interestingly, a 50 years old male patient with parasagittal meningioma and pathologically diagnosed as RDD (Patient No.5) harbored a pathogenic mutation in *BRCA1* (c.2157dupA) (**Table 3**). *BRCA1* plays a role in DNA double-strand break (DSB) repair via homologous recombination (16). It is also essential for the repair and restart of stalled and damaged DNA replication forks and protects them from nucleolytic attack and attrition (17, 18). Failure to remove DNA lesions or to restart stalled replication forks may lead to genome rearrangements or mutations that can cause cancer (16, 19). *BRCA1* is also a tumor suppressor gene that is involved in different cellular processes and regulates tumor development (20–22). Additionally, BRCA1 protein is a co-regulator of a broad range of transcriptional factors and plays a role in the epigenetic regulation of gene expression by chromatin remodeling (23, 24).

Mutations in *BRCA1* are highly associated with familial breast and ovarian cancer and are also the likely driver of a variety of sporadic cancers (25). While the association of *BRCA*1 mutations with breast and ovarian cancer risks is thoroughly studied, the occurrence of other cancers that are linked to *BRCA*1 mutations are limited. In the studies that focused on additional cancers associated with *BRCA1* mutations, increased risk has been reported. This included pancreatic, liver, esophagus, stomach, prostate, colorectal, uterine, and cervical cancers (26–29). The variant found in our study is a characterized frameshift mutation in breast and ovarian cancer (30). Durham et al, data showed mutations in genes involved in DNA mismatch repair pathways including *BRCA1, ERCC2, ATM*, and *LATS2* in 1/17 patients with RDD (8). Besides the pathogenic *BRCA1* mutation, Patient No.5 (**Table 3**) has two likely benign *ATM* mutations (c.1236-3dupT and c.3403-13dupA). We also identified a likely pathogenic variant of *LATS2* (c.1907_1908ins) in Patient No.2 (**Table 3**). Although further studies need to be conducted to investigate the link between these genetic lesions and RDD, this raises the question of whether the lack of DNA repair renders the cells prone to possibly oncogenic genome alterations that could lead to the development of the disease.

It is also important to acknowledge that because the tissue sampled was an RDD lesion, the *BRCA1* mutation identified in Patient No.5 might be somatic mutation specific to the histiocytes. However, his observation does not rule out a potential germline mutation, thus definitive determination of germline status would necessitate WES or targeted sequencing from a non-affected tissue source which was a limitation to our findings. To further elucidate the role of DNA repair gene mutations in RDD, future studies should examine the prevalence of *BRCA1* and other relevant genes in larger, well-characterized cohorts and integrate germline sequencing data to assess their contribution to disease development and progression

In addition to the previous mutations, we uncovered likely pathogenic *DNMT3A* (c.126dupG) variant in Patients No.1 and No.3 (**Table 3**). *DNMT3A* alterations are closely linked to myeloid and lymphoid malignancies (31).

Both patients did not have hematological cancer however, Patient No.3 (**Table 1**) is a female with breast carcinoma. Multiple studies suggest that DNMT3A is pivotal in breast oncogenesis and progression due to its DNA methylation function (32). Baraban et al, also revealed *DNMT3A* alteration along with MAPK related mutations in two patients with no history of a hematological neoplasm. Later diagnosis from bone marrow biopsy in one patient uncovered myelodysplastic syndrome, suggesting that it may have been present but undiagnosed at the time of the RDD diagnosis. A definitive analysis of the potential genetic link between RDD and hematological malignancy in the other patient was not possible, due to patient follow-up loss and the absence of a bone marrow biopsy (12). Similarly, bone marrow testing was unavailable for the patients in our study. This lack of follow-up precludes any conclusions regarding the presence of an underlying hematological malignancy.

Our analysis of the patients’ complete blood count (CBC) revealed no evidence suggestive of clonal hematopoiesis of indeterminate potential, clonal cytopenia of undetermined significance, or any other overt hematological abnormality. Specifically, the patient’s white blood cell count, red blood cell count, and platelet count remained within normal ranges throughout the follow-up period. There were no significant fluctuations or trends observed in the patient’s CBC parameters, and differential counts showed no evidence of abnormal cell populations. Based on the available CBC data, there is no indication that the *DNMT3A* mutation is associated with a clinically apparent hematological malignancy. However, CBC is not a substitute for a bone marrow biopsy and cannot definitively exclude the presence of hematological neoplasm. Ideally bone marrow analysis would provide a more comprehensive assessment of potential myeloid involvement in RDD patients with *DNMT3A* mutations. This highlights the need for long-term follow-up and bone marrow assessment in RDD patients harboring *DNMT3A* mutations to clarify whether these alterations contribute to disease pathogenesis, cancer predisposition, or represent incidental findings.

Our findings align with previous research indicating that RDD involves mutations in genes with diverse cellular functions. Durham et al, reported mutations in genes related to cell cycle regulation, transcriptional regulation, DNA mismatch repair, and the ubiquitin proteasome pathway (8). Similarly, our study identified VUS in *PDS5A*, *USP35*, and *CIC*, genes involved in cell cycle regulation, the ubiquitin proteasome pathway, and transcriptional regulation, respectively.

The recurrence of mutations in genes associated with these critical cellular processes suggests that dysregulation in these pathways may contribute to RDD pathogenesis. For instance, *PDS5A* plays a role in sister chromatid cohesion and DNA repair, and its disruption could lead to genomic instability (33). *USP35* is involved in regulating protein turnover through deubiquitination, and its alteration may affect cellular homeostasis (34). *CIC* is a transcriptional repressor implicated in various cancers, and its mutation could disrupt gene expression control (35).

For variants classified as VUS and likely benign according to ACMG guidelines, it indicates that these variants have relatively high allele frequencies in other populations, but definitive evidence of pathogenicity is currently lacking. Comparing allele frequencies of VUS variants in the Saudi population with other populations provides valuable insights. A high allele frequency in the Saudi population, mirroring observations in genes like *PDS5A*, supports the classification of VUS. However, a crucial point to consider is that *USP35* (c.2351C>T) and *CIC* (c.206G>A), both categorized as VUS, exhibit low allele frequencies within the Saudi population. This low frequency raises the possibility that these variants may not be mere polymorphisms but could potentially contribute to RDD pathogenesis in this population. This might be significant as these variants were identified in the same patient who also harbored a *DNMT3A* mutation. Adding to this complexity, we lack allele frequency data for the *DNMT3A* mutation in our Saudi population, further limiting our ability to assess its potential role in RDD within this specific genetic context. The low allele frequencies of *USP35* and *CIC*, coupled with the absence of *DNMT3A* allele frequency data in our population, strongly suggest the need for larger, population-specific studies to accurately interpret the significance of these variants in RDD pathogenesis, and to determine whether these low frequency VUS variants are pathogenic in our population or others.

While the functional consequences of these specific VUS require further investigation, their presence in our cohort and in previous studies suggests that a complex interplay of genetic alterations across various pathways may contribute to RDD development.

Although our study identified several genetic variants, the functional validation of these variants remains a significant limitation. Functional studies, such as *in vitro* or *in vivo* assays, are crucial to establish causality and to definitively determine the impact of these variants on cellular function and RDD pathogenesis. Without such functional data, we can only speculate on the potential roles of these variants in the disease process. Therefore, future studies should prioritize the functional characterization of identified variants to provide a more comprehensive understanding of their contributions to RDD.

In conclusion, our findings do not provide an implication that RDD is a clonal neoplasm driven by recurrent mutations in MAPK related genes. The mutated genes we found are closely related to multiple types of cancers. Some of which have DNA repair functions and others with DNA methylation and epigenetic alteration capabilities. The indication of oncogenic drivers on RDD classification as cancer is an area of debate, despite that it has features of transformed cells. Nonetheless, the mutations identified in our study and other studies raise the hypothesis that lesions in genome stability, and epigenetic regulation genes could be the driving force behind cellular transformations that lead to the development of RDD. The exact roles of these genes need further investigation in studies with a greater sample size of patients and functional analysis to explore the etiology and pathogenesis of RDD.

## Data Availability

The raw data supporting the conclusions of this article will be made available by the authors, without undue reservation.
